# Localization analysis of metabolites from complex biological samples-recent analytical technique of mass spectrometry imaging

**DOI:** 10.3389/fmolb.2023.1169449

**Published:** 2023-04-21

**Authors:** Qiang Yang, Ying Cai, Sifan Guo, Zhibo Wang, Shi Qiu, Aihua Zhang

**Affiliations:** ^1^ GAP Center and Graduate School, Heilongjiang University of Chinese Medicine, Harbin, China; ^2^ International Advanced Functional Omics Platform, Scientific Experiment Center, Hainan Medical University, Haikou, China

**Keywords:** mass spectrometry imaging, target, metabolomics, diagnosis, metabolite

Recently, some studies based on analytical methods and the application of mass spectroscopic imaging technology have been published in some academic journals. These studies have shown that mass spectrometry imaging (MSI) is the dominant technology applied in spatial localization, through scanning slices of biological samples and generating charged ions, obtaining the spatial distribution characteristics of various molecules via ion detectors, and performing data dimensionality reduction, statistical calculation, and visual analysis. These articles promote the development of MSI technology, which helps to efficiently and accurately mine MSI data to identify important molecular signatures, promote the application of MSI in clinical diagnosis, drug research and other fields, and lay the foundation for screening biomarkers of related diseases and elucidating the pharmacodynamic mechanism of drugs in organ microregions.

**FIGURE 1:**
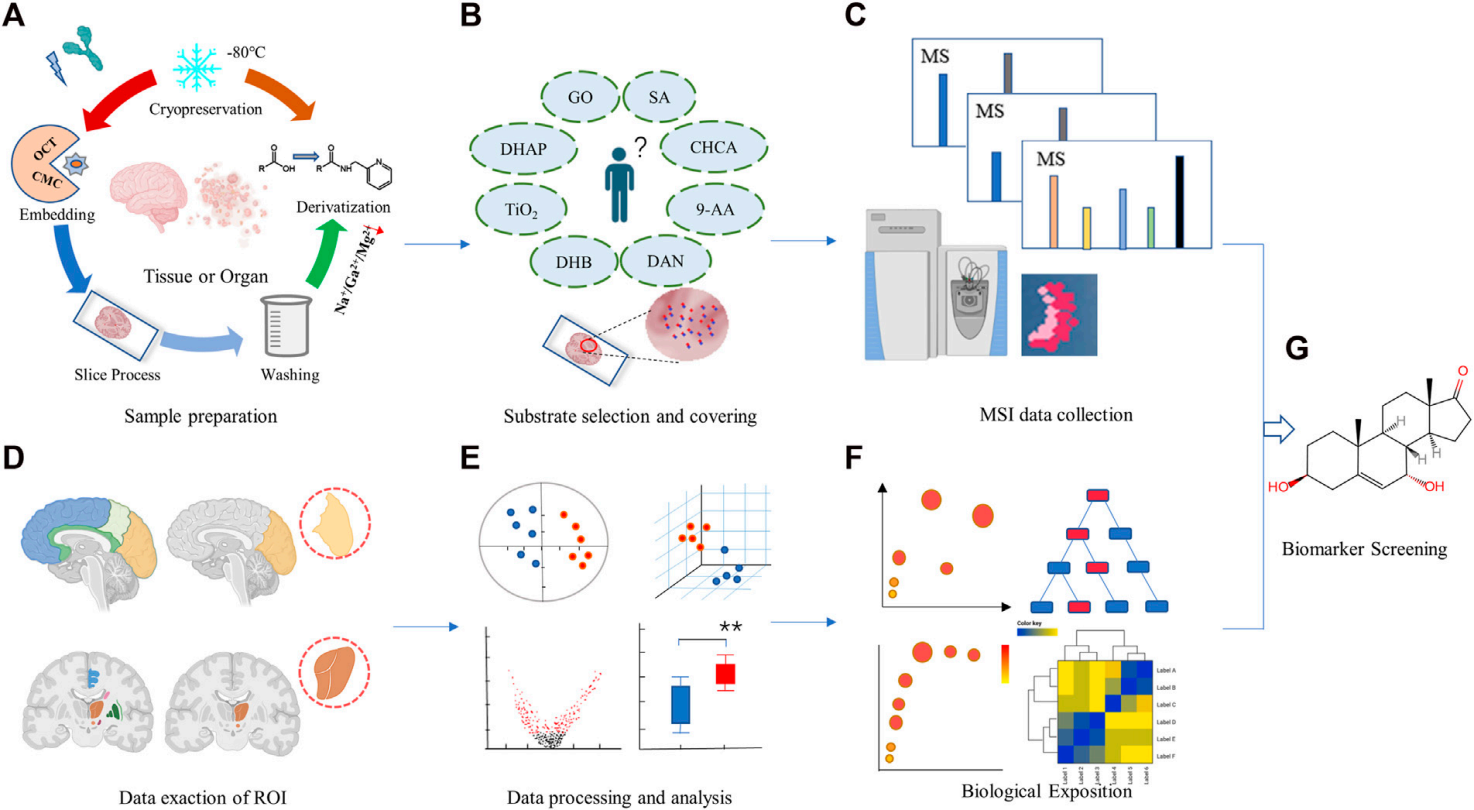
Analysis workflow for a typical spatial metabolomics. It mainly covers two parts: mass spectrometry imaging **(A–C)** and metabolomics studies **(D–F)**. Sample collection and pretreatment for mass spectrometry imaging **(A)**, selection and embedding of matrix **(B)**, data collection and analysis for mass spectrometry imaging **(C)**, Data exaction of ROI **(D)**, data processing and analysis **(E)**, and biological function description **(F)**. Biomarker screening and discovery **(G)**. The figure is created by BioRender and MetaboAnalyst.

Spatial metabolomics uses MSI technology to analyze the species, content and spatial distribution of metabolites in different tissues and organs, can overcome the bottleneck of spatial information loss in traditional metabolomics research (Qiu et al., 2023). Spatial metabolomics technology mainly relies on MSI and metabolomics technology. The specific process should include sample preparation, matrix selection and coverage, data acquisition and analysis, *etc.* (Figure 1). Luo et al. (2023) had identified a novel metabolic subtype of type 2b mitotic muscle fibers by cross-analyzing metabolomics analysis with MSI data. Fingerprint metabolites enriched include acylcarnitines, cyclic ADP-ribose, and thiamine pyrophosphate, *etc.*, which have anti-fatigue metabolic properties. Visualizing muscle fibers by using MSI may improve our understanding of muscle fiber remodeling under physiological and pathological conditions.

Linear dimensionality reduction methods, such as principal component analysis (PCA) and non-negative matrix factorization (NNMF), are widely used in MSI data analysis. In recent years, the non-linear dimensionality reduction method T-distributed random neighbor embedding has been widely used in omics data analysis. By means of dimensionality reduction, redundant information and noise information in high-dimensional space can be effectively removed, and the accuracy of image recognition can be improved. PCA, as one of the commonly used linear dimension reduction methods, uses the projection method to compress the high dimensional space to the low dimension, and maintains the characteristics that contribute the most to the variance of the data set. Non-linear dimensionality reduction is widely used in image data recognition problems to obtain better recognition results. As an unsupervised learning algorithm, NNMF is mainly used to extract useful features in multi-dimensional data, and has gradually become a widely accepted method for data processing in research fields such as biomedicine and image recognition. Recently, Abdelmoula et al. (2021) had developed a peak processing and data analysis method based on neural networks. This method is a molecular model used to predict atomic data by associating encoded features with primary data by minimizing the error between original and real data. This method is suitable for analyzing MSI low capacity data without preprocessing and peak extraction, and is generally applicable to different acquisition devices. Optimizing the data processing process and establishing a data clustering analysis scheme are crucial in MSI’s data analysis.

The brain is the central target of central nervous system diseases, and most central nervous system (CNS) drugs will produce pharmacological effects by acting on the brain. Therefore, mining the spatial information of drugs and related endogenous metabolites in the brain is of great significance for evaluating the drug efficacy and elaborating the relevant mechanism of action. Brain imaging techniques are used for the analysis of brain tissue structure, which cannot be comprehensively analyzed at the molecular level, and the types of substances that can be monitored are also limited. To be precise, a single metabolomics method can only reflect the average level of metabolites in a sample and lacks spatial distribution information. Liu D et al. (2022) studied the effects of olanzapine (OLZ) drugs on brain tissue and found that olanzapine exerted therapeutic effects or caused adverse reactions by regulating the metabolism of aspartate, glutamate and glycerophospholipid. Jin B et al. (2022) also investigated the spatiotemporal and dynamic distribution characteristics of endogenous metabolites in mouse brain microregions under YZG-331 intervention, and found the functional metabolite GABA related to the drug effect of YZG-331, and located the metabolic pathways “GABAergic synapse” and “histidine metabolism”. This study is helpful to interpret the mechanism of drug action of new drug candidates for the central nervous system and to find potential multi-targets, and to provide a visual analysis method for drug development.

MSI is more suitable for the identification of proteins, activation peptides, lipids and small biomolecules in tissue samples. These articles continue to improve MSI technology in the areas of sample pretreatment, data processing and functional analysis. MSI technology was used to analyze the spatial distribution of biological samples and reveal the molecular distribution mechanism of biological molecules. By combining MSI technology with multi-omics technology, spatial information and accurate quantification can be effectively correlated, which is of great significance for early diagnosis and prognosis of diseases, biomarker discovery, *etc.*

Although MSI has been widely used in recent years, there are still some limitations. For example, the imaging analysis of compounds with low abundance, low ionization efficiency, and easy interference by matrix peaks is poor, and the method of dimensionality reduction and clustering analysis of high-dimensional MSI data is not immaculate. Future work should focus on developing powerful statistical and deep learning methods to rapidly extract comprehensive molecular information with diagnostic capabilities, and developing new strategies for various qualitative and quantitative MSI based on simulated tissue models or standard deposition. The development of MSI technology has broad application prospects in the fields of omics analysis, precision medicine, toxicology and drug metabolism.
